# Acute Effect of Two Different Hand Exercises on Vessel Size in Patients Undergoing Arteriovenous Fistula Creation

**DOI:** 10.3400/avd.oa.24-00136

**Published:** 2025-04-17

**Authors:** Yuthapong Wongmahisorn, Pong Kanchanasuttirak, Waigoon Stapanavatr, Yupadee Fusakul

**Affiliations:** 1 Department of Surgery, Faculty of Medicine Vajira Hospital, Navamindradhiraj University, Bangkok, Thailand; 2 Department of Physical Medicine and Rehabilitation, Faculty of Medicine Vajira Hospital, Navamindradhiraj University, Bangkok, Thailand

**Keywords:** arteriovenous fistula, blood flow rate, hand exercises, tourniquet-like compression, venous diameter

## Abstract

**Objectives:** We primarily aimed to compare the acute effects of hand-squeezing exercises, with and without tourniquet-like compression, on vessel size 5 minutes post-exercise in patients after arteriovenous fistula (AVF) creation. The secondary aim was to assess differences in AVF blood flow rates between the 2 interventions.

**Methods:** A randomized study was conducted at a university hospital in Bangkok, Thailand, from October 2018 to September 2023. Seventy-eight participants, 2 weeks post-first-time autogenous AVF creation, were randomized into 2 groups: a non-compression group (n = 39) performing 5 minutes of hand-squeezing exercises and a compression group (n = 39) performing the same exercises with tourniquet-like compression. Ultrasound measured venous diameter and blood flow rates pre- and post-exercise.

**Results:** Both groups showed increased venous diameter, but the difference between the groups was not statistically significant (mean difference: 0.18 mm with compression vs. 0.12 mm without; P = 0.489). Blood flow rates increased significantly in the compression group compared to the non-compression group (mean difference: 171.49 vs. 24.44 mL/min; P = 0.002).

**Conclusion:** Hand-squeezing exercises with tourniquet-like compression significantly improved AVF blood flow rates acutely, supporting its potential to enhance AVF maturation. Further research is needed to assess long-term benefits.

## Introduction

The arteriovenous fistula (AVF), first introduced by Brescia et al. in 1966,^1)^ remains the gold standard for hemodialysis access in patients with end-stage renal disease (ESRD). This surgical procedure creates a direct connection between arterial and venous vessels, offering superior durability and safety compared to arteriovenous grafts or central venous catheters.^2–4)^ Both the National Kidney Foundation Kidney Disease Outcomes Quality Initiative (NKF KDOQI) and the American Society for Vascular Surgery recognize the AVF as the preferred choice for long-term hemodialysis.^5,6)^

Successful AVF function depends on a critical maturation period of 4–6 weeks or longer, during which vessels must achieve sufficient size and stability for cannulation. However, maturation fails in 20%–50% of cases due to various factors,^7–10)^ including patient characteristics, surgical technique, and postoperative care.^11–14)^ To enhance AVF maturation, the NKF KDOQI guidelines recommend postoperative hand exercises,^5)^ such as isometric hand gripping or light tourniquet use.^15–17)^ One study reported that combining hand exercises with a tourniquet enhanced vessel size, blood flow, and clinical maturation rates compared to hand-squeezing exercises alone after 2 weeks.^18)^ Conversely, another study found no improvement in venous diameter or blood flow 3 months post-surgery when an upper arm tourniquet was added to handgrip exercises.^19)^

To date, only 1 study has investigated the acute effects of hand-squeezing exercises on vessel size, reporting a 9.3% increase in fistula diameter after a 5-minute hand-squeezing exercise in patients approximately 2.8 months post-AVF surgery.^20)^ However, the acute effects of combining hand-squeezing exercises with tourniquet use remain unknown.

To address this knowledge gap, we designed a study to examine whether combining hand-squeezing exercises with a tourniquet-like compression technique could acutely increase vessel size and blood flow within 5 minutes. Our modified method involves using the non-surgical hand to apply pressure 12–15 cm proximal to the AVF site while squeezing a ball with the AVF-affected hand, replacing the traditional tourniquet. This self-controlled technique effectively targets veins while minimizing the risk of venous thrombosis associated with blood flow stagnation from prolonged high-pressure tourniquet application. Our primary aim was to compare the acute effects of hand-squeezing exercises, with and without a tourniquet-like compression technique, on venous diameter 5 minutes post-exercise in patients 2 weeks after AVF creation. The secondary aim was to assess differences in AVF blood flow rates between these 2 exercise interventions.

## Materials and Methods

### Study design and setting

This randomized study was conducted at the Faculty of Medicine Vajira Hospital, Bangkok, Thailand, from October 2018 to September 2023. It was part of a research project evaluating the effects of 2 different hand exercises on the outcomes of AVF creation for hemodialysis. The study specifically examined the acute effects of hand-squeezing exercises in adult patients with ESRD attending the vascular surgery clinic for their first-time autogenous AVF creation. The trial adhered to the Consolidated Standards of Reporting Trials (CONSORT) guidelines. Ethical approval was granted by the Institutional Review Board (certificate of approval no. 76/2561; approved on May 31, 2018). The trial was registered with the Thai Clinical Trials Registry on September 17, 2018 (registration number TCTR20180917005). All participants provided written informed consent prior to enrollment.

### Participants

Patients referred for first-time autogenous AVF creation were screened for eligibility. Participants were eligible for inclusion if they were 18 years of age or older, scheduled for their first-time autogenous AVF surgery (either radiocephalic or brachiocephalic), had good functionality in both hands and arms, and were able to communicate in Thai. Participants were excluded from the study if they had joint disease or nerve abnormalities in the hand on the side of the planned AVF surgery; medical conditions causing hand weakness, such as stroke, peripheral neuropathy, radiculopathy, or peripheral arterial disease; swelling or inflammation in the surgical area; inability to follow exercise guidelines; or refusal to participate.

### Sample size

The sample size estimation was based on a pilot study involving 6 individuals, which assessed venous diameter 5 minutes after hand-squeezing exercises. The study compared the results with and without compression applied to the upper arm, revealing mean venous diameters of 3.63 ± 0.45 and 4.02 ± 0.52 mm, respectively. Using these values, a pooled variance of 0.49, a significance level of 0.05, and a power of 80%, the minimum required sample size was calculated to be 50 participants, with 25 in each group. However, as this study was part of a larger investigation examining the effects of these exercise methods on vessel parameters by 10 weeks post-surgery, the final sample size included 78 participants.

### AVF creation

At our institution, patients with ESRD undergoing first-time AVF creation are routinely evaluated for vascular anatomy suitability. Preoperative assessments include physical examinations and ultrasound evaluations, as outlined in a previous publication.^21)^ Potentially eligible patients were initially approached and counseled at the vascular surgery clinic, where they were provided with a comprehensive study information sheet. Patients who expressed willingness to participate proceeded with their scheduled surgical operation.

Each AVF creation was performed by 1 of the 3 investigators (YW, PK, or WS), all of whom are experienced vascular surgeons. The procedure involved creating an anastomosis between the end of the cephalic vein and the side of either the radial artery (radiocephalic anastomosis) or the brachial artery (brachiocephalic anastomosis), depending on the surgeon’s discretion and the feasibility of the vessels. Details of the surgical techniques have been described previously.^21)^

### Randomization and exercise protocol

Participants were scheduled for a follow-up visit 2 weeks post-surgery. On the designated date, participants were randomly assigned in a 1:1 ratio to 2 groups: a non-compression group performing hand-squeezing exercises alone, and a compression group performing hand-squeezing exercises with compression applied 12–15 cm proximal to the AVF site on the affected hand. Randomization was executed using a computer-generated sequence. A research assistant uninvolved in the study’s primary investigations prepared sealed, sequentially numbered, opaque envelopes containing group assignments, which were opened immediately on the day of the follow-up visit after surgery. Allocation remained concealed from the investigators. Due to the intervention’s inherent nature, participant blinding was not feasible.

**Figure 1:**
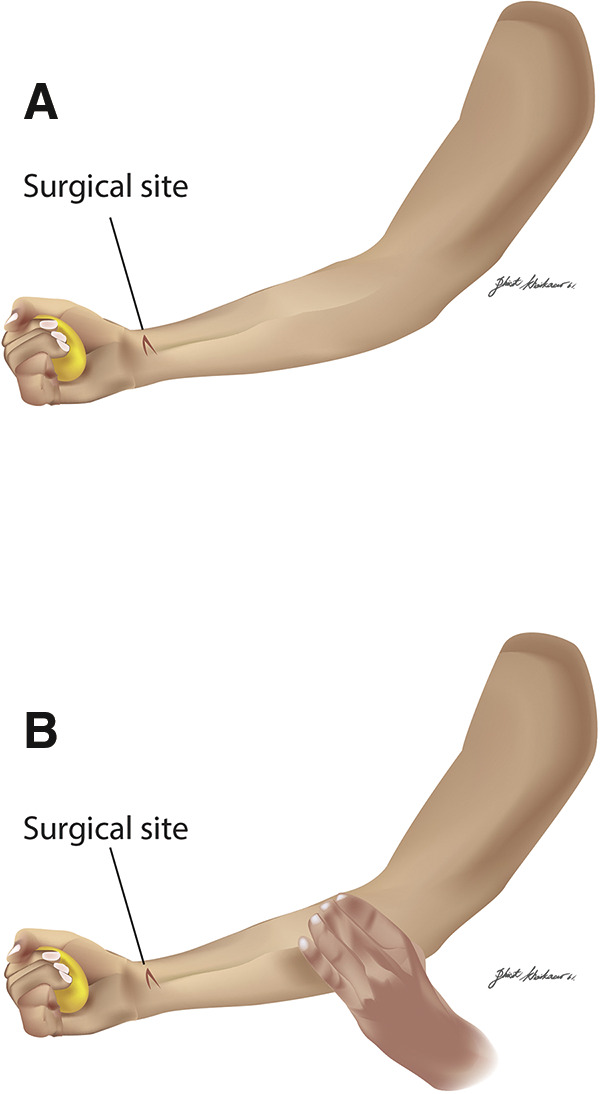
Fig. 1 Illustration of ball-squeezing exercises: (**A**) without compression and (**B**) with compression

In the non-compression group, participants received instructions from a nurse assistant to perform hand-squeezing exercises. While seated, they rested their operated arm on the same-side thigh with the palm facing upward (**Fig. 1A**). Using the operated hand, participants squeezed the ball with maximum force, holding each squeeze for 5 seconds (counting from 1 to 5), followed by a 2-second release (counting from 1 to 2). This sequence was repeated continuously for 5 minutes.

In the compression group, participants received instructions from a nurse assistant to combine hand-squeezing exercises with compression applied proximal to the surgical site on the operated arm. While seated, they rested their operated arm on the same-side thigh with the palm facing upward. Participants used the operated hand to squeeze a ball, adhering to the same intensity and timing protocol as the non-compression group. Simultaneously, they applied compression approximately 12–15 cm proximal to the AVF surgical site in rhythm with the ball-squeezing. Compression was applied using the non-operated hand, positioned palm-down, with 4 fingers (index to pinky) pressing the vein and the thumb placed underneath the operated arm (**Fig. 1B**). This combined exercise sequence was also repeated continuously for 5 minutes.

### Ultrasound measurements

Ultrasound assessments were performed on the superficial vein 3 cm from the anastomosis site to evaluate parameters at baseline (pre-exercise) and immediately after the 5-minute hand-squeezing exercises (post-exercise). The assessed parameters included venous diameter, vein wall thickness, skin-to-vein distance, and blood flow rate. Doppler ultrasound was performed using a standard 60° insonation angle.

All assessments were conducted using a GE LOGIQ E9 ultrasound machine (GE Healthcare, Chicago, IL, USA) with an 8–18 MHz linear hockey stick probe. Each parameter was measured 3 times within 1–2 minutes, and the average was recorded for analysis. A single experienced vascular surgeon, blinded to the participants’ allocation groups, performed all examinations. The intraclass correlation coefficient for the ultrasound measurements was 0.95, indicating excellent intra-assessor reliability.

### Data collection and outcome measures

Data were collected by a nurse assistant who was blinded to the participants’ allocation groups. The collected information included demographic characteristics, smoking status, comorbid conditions, current medications, the type of the AVF, the arm on which the AVF was performed, the participant’s dominant hand, and ultrasound parameters. The primary exposure variable was the exercise method, while the outcome measures were venous diameter and AVF blood flow rate.

### Statistical analyses

Patient characteristics are summarized as mean ± standard deviation or frequency (percentage), as appropriate. The Student’s t-test was used to compare continuous variables between groups, while categorical variables were analyzed using the chi-square test or Fisher’s exact test. Paired t-tests were used to compare pre- and post-exercise changes (mean differences) in vessel parameters within each group. Between-group mean differences were analyzed using linear regression, with adjustments for potential associated factors such as sex, baseline blood flow rates, and dominant hand. All data were analyzed using IBM SPSS Statistics for Windows, Version 28.0 (IBM Corporation, Armonk, NY, USA). Statistical significance was set at P < 0.05.

## Results

### Participant flow and baseline characteristics

**Figure 2:**
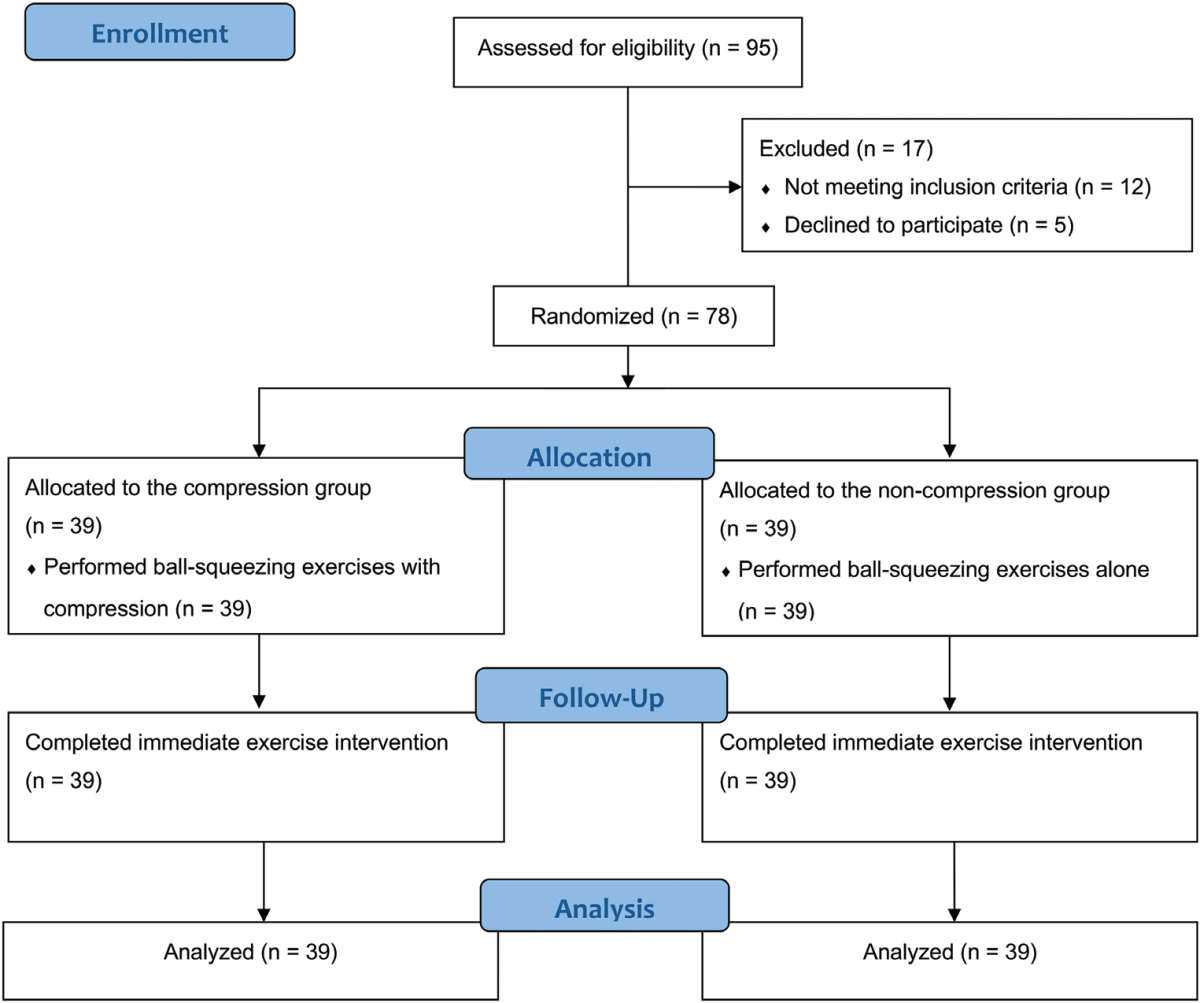
Fig. 2 CONSORT flowchart of patient recruitment and study flow. CONSORT: Consolidated Standards of Reporting Trials

A total of 95 patients were screened for eligibility, with 17 excluded due to not meeting inclusion criteria or declining participation (**Fig. 2**). Seventy-eight participants were ultimately enrolled and randomized: 39 in the non-compression group (hand-squeezing exercises alone) and 39 in the compression group (hand-squeezing exercises with compression). Baseline characteristics, including age, sex, body mass index, smoking status, comorbid conditions, current medications, AVF type, side, and dominant hand, were comparable between the 2 groups (**Table 1**).

**Table:** Table 1 Participant characteristics

	Non-compression group (n = 39)	Compression group (n = 39)	P value
Age (years)	57.5 ± 13.8	55.4 ± 13.6	0.505
Male	23 (59.0)	22 (56.4)	0.819
BMI (kg/m^2^)	25.0 ± 4.9	23.9 ± 4.7	0.322
Current smoking	3 (7.7)	2 (5.1)	0.644
Comorbid conditions			
DM	28 (71.8)	24 (61.5)	0.337
Hypertension	36 (92.3)	34 (87.2)	0.455
Dyslipidemia	30 (76.9)	23 (59.0)	0.089
Current medication			
Antiplatelet drugs	12 (30.8)	9 (23.1)	0.444
Anticoagulants	12 (30.8)	6 (15.4)	0.107
Type of AVF			0.241
Radiocephalic	12 (30.8)	17 (43.6)	
Brachiocephalic	27 (69.2)	22 (56.4)	
AVF side			0.496
Right	17 (43.6)	20 (51.3)	
Left	22 (56.4)	19 (48.7)	
Dominant hand			1.000
Right	38 (97.4)	38 (97.4)	
Left	1 (2.6)	1 (2.6)	

Data are presented as the mean ± SD or n (%). AVF: arteriovenous fistula; BMI: body mass index; DM: diabetes mellitus; SD: standard deviation

### Primary and secondary outcomes

**Table 2** presents the ultrasound measurements of vessel parameters before and after exercise for both groups. At baseline, venous diameter, vein wall thickness, skin-to-vein distance, and AVF blood flow rate were similar between the groups.

**Table:** Table 2 Comparisons of vessel parameters before and after exercise between the two exercise groups

Parameter	Non-compression group (n = 39)			P value^a^	Compression group (n = 39)			P value^a^	P value^b^
	Pre-exercise	Post-exercise	Mean difference		Pre-exercise	Post-exercise	Mean difference		
Venous diameter (mm)	5.56 (5.24–5.89)	5.68 (5.34–6.03)	0.12 (0.02 to 0.21)	0.016	5.37 (5.04–5.70)	5.55 (5.20–5.91)	0.18 (0.08 to 0.29)	0.001	0.489
Vein wall thickness (mm)	0.43 (0.41–0.46)	0.45 (0.42–0.47)	0.02 (0.00 to 0.03)	0.442	0.44 (0.41–0.47)	0.43 (0.39–0.47)	–0.01 (–0.04 to 0.01)	0.335	0.201
Skin-to-vein distance (mm)	2.76 (2.32–3.22)	2.68 (2.24–3.13)	–0.08 (–0.21 to 0.04)	0.083	2.77 (2.27–3.27)	2.76 (2.25–3.27)	–0.01 (–0.14 to 0.12)	0.845	0.418
Blood flow rate (mL/min)	791.23 (677.66–904.80)	815.67 (685.80–945.53)	24.44(–39.20 to 88.07)	0.163	802.28 (704.66–899.90)	973.77 (849.60–1097.94)	171.49 (102.34 to 240.63)	<0.001	0.002

Data are presented as mean (95% confidence interval).

^a^Changes between pre- and post-exercise within a group were analyzed using the paired t-test.

^b^Mean differences between exercise groups were compared using linear regression, adjusting for sex, baseline blood flow rates, and dominant hand.

**Figure 3:**
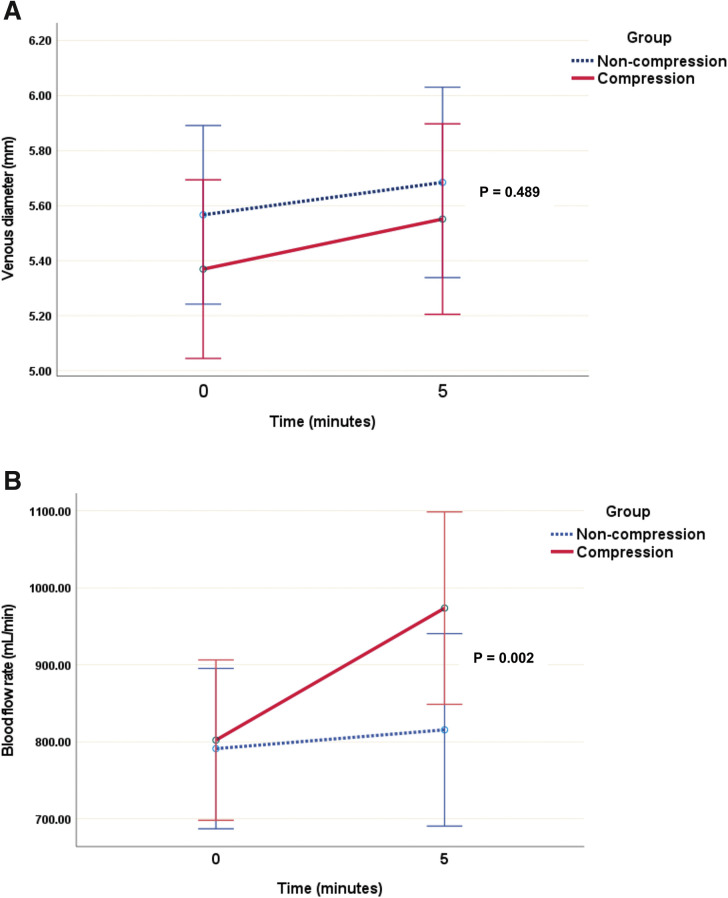
Fig. 3 Changes in (**A**) venous diameter and (**B**) blood flow rate before and after exercise between the 2 exercise groups.

Both hand-squeezing exercises, with and without tourniquet-like compression, acutely increased venous diameter (**Fig. 3A**), but the difference between methods was not statistically significant (0.18 vs. 0.12 mm without; P = 0.489). In contrast, blood flow rate changes differed significantly (**Fig. 3B**), with compression yielding a greater increase (171.49 vs. 24.44 mL/min; P = 0.002). Subgroup analysis revealed that this effect was significant only in participants with brachiocephalic anastomosis (**Supplementary Tables 1 and 2**). Additionally, linear regression analysis showed that sex was significantly associated with changes in blood flow rate (mean difference: 145.44 mL/min in males vs. 33.21 mL/min in females; P = 0.013), while baseline blood flow rates and dominant hand showed no significant associations. Neither exercise method had a significant acute effect on vein wall thickness or skin-to-vein distance.

## Discussion

This randomized study demonstrated that both hand-squeezing exercises, with and without tourniquet-like compression, acutely and significantly increased venous diameter. However, the increases were modest (0.18 vs. 0.12 mm; 3% vs. 2%) and not significantly different between the groups. In contrast, the compression technique led to a significantly greater improvement in blood flow rates compared to exercises alone (171.49 vs. 24.44 mL/min, representing increases of 21% vs. 3%).

Our findings aligned with previous research on post-AVF exercise interventions. The acute increase in venous diameter was consistent with Oder et al.’s report of a 9.3% increase following 5-minute hand-squeezing exercises,^20)^ reinforcing the role of isometric hand exercises in vessel dilation. Notably, our study provides novel evidence that incorporating compression into the exercise protocol significantly improved blood flow rates. This finding aligns with Salimi et al., who reported increased vessel size, higher blood flow rates, and improved clinical maturation at 2 weeks when hand-squeezing exercises were combined with a tourniquet.^18)^ In contrast, Chen et al. found that adding an upper arm tourniquet to handgrip exercises did not enhance venous diameter or blood flow 3 months after surgery.^19)^ These discrepancies likely stem from differences in assessment timing, exercise intensity, compression techniques, and AVF types. While our study focused on acute effects after a 5-minute exercise, Salimi et al. and Chen et al. assessed outcomes over longer periods (2 weeks and 3 months, respectively). Additionally, our compression technique involved manual pressure 12–15 cm proximal to the AVF site, whereas Salimi et al. and Chen et al. used a tourniquet. Furthermore, most of our participants and all of Salimi et al.’s cohort had brachiocephalic AVFs, whereas Chen et al.’s study exclusively involved radiocephalic AVFs.

Our subgroup analysis further supports these distinctions, revealing a significant effect of compression on blood flow in the brachiocephalic cohort but not in the radiocephalic cohort. This finding aligns with Salimi et al.’s conclusion that compression benefits brachiocephalic AVFs while also corroborating Chen et al.’s observation that compression has no effect on radiocephalic AVFs.

Despite randomization, the distribution of brachiocephalic AVFs differed between groups (69% in the non-compression group vs. 56% in the compression group). Given the higher baseline blood flow rates in brachiocephalic AVFs compared to radiocephalic AVFs (853.26 vs. 700.93 mL/min; P = 0.045), this imbalance could have influenced blood flow changes. To account for this, linear regression analysis was performed with baseline blood flow as a covariate. The results confirmed that exercises with compression significantly increased blood flow, independent of baseline values.

The enhanced blood flow observed with the tourniquet-like compression technique can be explained by several physiological mechanisms. Proximal compression restricts venous outflow, leading to venous pooling distal to the compression site. This pooling, combined with hand-squeezing exercises, triggers vascular responses: endothelial cells produce nitric oxide due to increased wall tension and shear stress^22)^; vessel wall mechanoreceptors promote vasodilation^23)^; and metabolites accumulate locally.^24)^ Upon compression release, the pooled blood induces a rapid flush effect, enhancing reperfusion, vascular remodeling,^25)^ pro-inflammatory mediator clearance, and oxygen delivery to vascular tissues. This cyclical pattern of compression and release fosters optimal conditions for vascular adaptation, explaining the superior blood flow improvements.

The differing effects on blood flow rates and venous diameter suggest that the compression technique primarily affects flow dynamics rather than causing immediate structural changes. This may be due to the rapid flush effect from rhythmic compression and decompression, which temporarily enhances blood flow. However, given the acute 5-minute intervention, the initial vessel enlargement likely decreased as blood flow diminished after the exercise ended. Consequently, the changes in venous diameter were modest, underscoring that structural remodeling requires sustained exercise interventions.

These mechanistic insights have practical implications for optimizing exercise protocols. The improvement in blood flow with compression underscores its potential value during the critical early maturation period, when establishing adequate flow is key to AVF development. A self-administered compression technique offers advantages over traditional tourniquets, improving patient compliance while maintaining effectiveness. By combining compression with hand-squeezing exercises postoperatively, clinicians can optimize AVF blood flow and potentially improve maturation outcomes. This noninvasive, cost-effective, and teachable method can enhance AVF functionality and reduce the risk of AVF maturation failure, minimizing the need for additional interventions.

Further research should explore the long-term effects of this combined exercise technique on AVF maturation rates and clinical success. Investigating whether the acute blood flow improvements observed in this study translate into sustained improvements over weeks or months would be valuable. Future studies could also examine the optimal duration, frequency, and intensity of exercises to maximize benefits and assess the applicability of this technique to other populations, such as those with challenging vascular anatomy or recurrent AVF creation.

The study’s strengths include its randomized controlled design, robust methodology, and the use of ultrasound measurements performed by a blinded expert, ensuring high reliability of results. Additionally, the study fills a critical gap in the literature by focusing on the acute effects within a specific time frame post-surgery.

However, several limitations should be acknowledged. First, this study focused on the immediate physiological responses to exercise without assessing its prolonged effects. Thus, the duration of hand exercise effects after cessation remains unknown. Additionally, we examined only AVF-associated superficial veins, excluding deep veins, which are less affected by external compression due to their location beneath the fascia. The compression technique also relied on patient self-administration, which may have introduced variability. Furthermore, while proximal compression during hand exercises may enhance blood flow, repeated use could exert mechanical stress on venous valves, potentially leading to distal reflux over time. Although our study examined immediate effects, its long-term impacts on venous valve function remain uncertain. Further research is needed to evaluate potential risks to venous integrity. Lastly, the single-center design limits the generalizability of our findings. Future multicenter trials with standardized training protocols could help address this limitation.

## Conclusion

This study provides new evidence that combining hand-squeezing exercises with a tourniquet-like compression technique significantly enhances blood flow immediately post-exercise, offering a promising strategy to improve AVF outcomes. The intervention is simple, cost-effective, and patient-driven, making it a practical addition to postoperative care protocols. However, further research is needed to establish its long-term benefits and refine its application for broader clinical use.

## Declarations

### Acknowledgments

We would like to thank Dr. Chadakarn Phaloprakarn for her valuable statistical advice and assistance with manuscript preparation. Additionally, all figures in this paper were edited by Mr. Phisit Khaikaew, a medical illustrator at our institution.

### Funding

This work was supported by the Navamindradhiraj University Research Fund.

### Disclosure statement

The authors have no conflict of interest to disclose.

### Author contributions

Study conception: all authors

Data collection: YW

Analysis: YW, PK

Investigation: YW

Manuscript preparation: YW, PK

Funding acquisition: YW

Critical review and revision: all authors

Final approval of the article: all authors

Accountability for all aspects of the work: all authors.

## Supplementary Information

Supplementary Table 1 Comparisons of venous diameter and blood flow rate before and after exercise in participants with radiocephalic anastomosis

Supplementary Table 2 Comparisons of venous diameter and blood flow rate before and after exercise in participants with brachiocephalic anastomosis
