# Supplementary material for: Acute Effect of Two Different Hand Exercises on Vessel Size in Patients Undergoing Arteriovenous Fistula Creation
Source: Ann Vasc Dis. 2025 Apr 17;18(1):24-00136. doi: 10.3400/avd.oa.24-00136 (PMC12014282; doi:10.3400/avd.oa.24-00136)
Supplement: Supplementary Table 1 — Comparisons of venous diameter and blood flow rate before and after exercise in participants with radiocephalic anastomosis [file avd-18-1-24-00136-s01.pdf]

Supplementary Table 1 Comparisons of venous diameter and blood flow rate before and after exercise in participants with radiocephalic anastomosis (n = 29)

Parameter	Non-compression group (n = 12)				Compression group (n = 17)				
	Pre-exercise	Post-exercise	Mean difference	P value[a]	Pre-exercise	Post-exercise	Mean difference	P value[a]	P value[b]
Venous diameter (mm)	5.14 (4.70–5.59)	5.13 (4.72–5.53)	–0.02 (–0.16 to 0.13)	0.806	4.90 (4.44–5.36)	5.01 (4.58–5.45)	0.11 (0.02 to 0.20)	0.017	0.103
Blood flow rate (mL/min)	622.75 (390.49–855.01)	754.42 (476.30–1032.53)	131.66 (–40.53 to 303.87)	0.121	756.12 (587.87–924.37)	896.12 (737.31–1062.93)	194.00 (62.21 to 325.79)	0.007	0.098

Data are presented as mean (95% confidence interval).

[a]Changes between pre- and post-exercise within a group were analyzed using the paired t-test.

[b]Mean differences between exercise groups were compared using linear regression, adjusting for sex, baseline blood flow rates, and dominant hand.

Supplementary Table 2 Comparisons of venous diameter and blood flow rate before and after exercise in participants with brachiocephalic anastomosis (n = 49)

Parameter	Non-compression group (n = 27)				Compression group (n = 22)				
	Pre-exercise	Post-exercise	Mean difference	P value[a]	Pre-exercise	Post-exercise	Mean difference	P value[a]	P value[b]
Venous diameter (mm)	5.75 (5.33–6.18)	5.93 (5.48–6.38)	0.18 (0.06 to 0.30)	0.005	5.73 (5.30–6.17)	5.97 (5.48–6.46)	0.24 (0.06 to 0.41)	0.011	0.738
Blood flow rate (mL/min)	825.37 (693.41–957.33)	842.89 (689.16–996.62)	17.52 (–69.76 to 104.79)	0.683	887.50 (769.23–1005.77)	992.05 (829.64–1154.45)	104.55 (17.76 to 191.33)	0.021	0.011

Data are presented as mean (95% confidence interval).

[a]Changes between pre- and post-exercise within a group were analyzed using the paired t-test.

[b]Mean differences between exercise groups were compared using linear regression, adjusting for sex, baseline blood flow rates, and dominant hand.
